# Reviewing the Etiologic Agents, Microbe-Host Relationship, Immune Response, Diagnosis, and Treatment in Chromoblastomycosis

**DOI:** 10.1155/2021/9742832

**Published:** 2021-11-01

**Authors:** Luiz Felipe Domingues Passero, Italo Novais Cavallone, Walter Belda

**Affiliations:** ^1^São Paulo State University (UNESP), Institute of Biosciences, São Vicente, Brazil; ^2^São Paulo State University (UNESP), Institute for Advanced Studies of Ocean, São Vicente, Brazil; ^3^Dermatology Department, University of São Paulo, Medical School, Clinics Hospital, São Paulo, Brazil; ^4^Laboratory of Pathology of Infectious Diseases, Medical School, University of São Paulo, São Paulo, Brazil

## Abstract

Chromoblastomycosis (CBM) is a neglected human disease, caused by different species of pigmented dematiaceous fungi that cause subcutaneous infections. This disease has been considered an occupational disease, occurring among people working in the field of agriculture, particularly in low-income countries. In 1914, the first case of CBM was described in Brazil, and although efforts have been made, few scientific and technological advances have been made in this area. In the field of fungi and host cell relationship, a very reduced number of antigens were characterized, but available data suggest that ectoantigens bind to the cell membrane of host cells and modulate the phagocytic, immunological, and microbicidal responses of immune cells. Furthermore, antigens cleave extracellular proteins in tissues, allowing fungi to spread. On the contrary, if phagocytic cells are able to present antigens in MHC molecules to T lymphocytes in the presence of costimulation and IL-12, a Th1 immune response will develop and a relative control of the disease will be observed. Despite knowledge of the resistance and susceptibility in CBM, up to now, no effective vaccines have been developed. In the field of chemotherapy, most patients are treated with conventional antifungal drugs, such as itraconazole and terbinafine, but these drugs exhibit limitations, considering that not all patients heal cutaneous lesions. Few advances in treatment have been made so far, but one of the most promising ones is based on the use of immunomodulators, such as imiquimod. Data about a standard treatment are missing in the medical literature; part of it is caused by the existence of a diversity of etiologic agents and clinical forms. The present review summarizes the advances made in the field of CBM related to the diversity of pathogenic species, fungi and host cell relationship, antigens, innate and acquired immunity, clinical forms of CBM, chemotherapy, and diagnosis.

## 1. Introduction

Chromoblastomycosis (CBM) is a chronic granulomatous and suppurative skin infection and is classified as a subcutaneous mycosis caused by pigmented dematiaceous fungi, prevalent in tropical and subtropical areas of the world. The fungi live in the soil and can be associated with plants; however, other etiologic agents of CBM have already been identified in other environments. In general, the fungus accesses human skin through sharp material injuries, allowing the entrance of the hyphae through the skin [1].

CBM is considered an occupational disease, which occurs primarily among farm laborers, palm tree and babassu coconut harvesters, lumberjacks, and agricultural product traders. Furthermore, the most vulnerable people generally work with conventional methods of growing and harvesting plants, making it easy for humans to come into contact with the etiologic agents. Furthermore, this vulnerable group of humans sometimes works without any type of protection, such as gloves, shoes, or garments, that may prevent skin injuries and subsequent infection [2].

It is important to highlight at least two important notes: (1) people at risk of contracting CBM work in tropical countries, where the temperature can be higher than 40°C in summer, and people refuse to wear protective equipment during the day (shoes, gloves, garment, etc.), even though they know that this kind of prophylactic measure could prevent different types of diseases and (2) vulnerable people generally live in low-income countries [3] and sometimes live far away from medical services, and once infected, they do not seek medical attention. In addition, treatment takes too long, and some patients abandon the therapy. The socioeconomic condition of the patients is another concern, as sometimes patients cannot afford protective equipment or even drugs to treat CBM.

With respect to the treatment of CBM, it has been considered a real challenge for physicians, researchers, and, above all, patients. There are few therapeutic options that are based mainly on couple options, such as itraconazole and terbinafine; physical approaches, such as surgery and cryotherapy, are interesting options; however, some factors, such as the type, extension, and place of the lesions, may limit the use of such methods. Furthermore, extensive randomized or comparative clinical trials on the best therapeutic options are rarely conducted in the medical literature, which could guide physicians to the best therapeutic options. Nowadays, it is necessary to consider the healthy state of the patient, comorbidities, socioeconomic conditions, and adherence to the proposed therapy [4]. Furthermore, few studies have characterized the mycotic action of new molecules on etiologic agents of CBM [5, 6], and most published studies show the activity of some drugs in CBM that are already used for other medical purposes; therefore, there is a huge gap in the development of specific drugs for the treatment of CBM. Furthermore, until now, no vaccines have been characterized to protect people from infection.

On 2017, CBM was added to the list of neglected tropical diseases of the World Health Organization [7], because most cases occur in low-income tropical countries and affect people living in poor communities, and few therapeutic options are available, which in fact are ineffective and highly toxic to patients. The present review is aimed at summarizing the main advances made in the last few years in the field of biodiversity of etiologic agents, physiopathology, immunology, and CBM treatment.

## 2. History of CBM

The first description of CBM was attributed to Maximilliano Willibaldo Rudolph in 1914 in patients living in Minas Gerais state, Brazil. Rudolph described in detail the acral-localized disease, designed in Portuguese as ‘Figueira' or ‘fig tree,' and contained data on the isolation of pigmented fungi in two of four studied patients [8]. However, a verrucous skin disease with a predominance of a brownish round-shaped microbe (muriform cells) was recorded in 1911 in Brazil, but it was associated with blastomycosis and was entitled black blastomycosis [9].

Lane and Medlar, in individual publications, reported the first case of CBM in the United States of America, in a patient with warty violet plaque in the buttocks and identified the *Phialophora verrucosa* species [10, 11]. The first case of CBM characterized outside the American continent described CBM in an Algerian patient [12], and the second case in the United States was described by Wilson and colleagues in 1933 [13].

The term CBM was first used by Terra and collaborators in 1922 to differentiate the mycotic disease found in Brazil from those related to verrucous dermatitis [14, 15]. The name CBM was recommended by Moore and Almeida in 1936 [16], and rapidly, all fungal infections caused by pigmented fungi were named CBM, including disseminated and cerebral fungal infections. In 1975, Ajello created the term phaeohyphomycosis to gather systemic and cutaneous fungal diseases that were different from CBM [17]. In this case, the clinical and histopathological features of the lesions are fundamental factors in the differentiation of the type of mycosis, as muriform cells are found in the histological section of the skin in cases of CBM while hyphae and yeast are found in phaeohyphomycosis [17, 18].

Since the description of the first case of CBM, different clinical forms have been described and a diversity of pathogenic fungi species have been incriminated in the development of the disease; these species live in organic material in soil, plants, and aquatic environments; thus, in addition to being considered a new disease, there is an epidemiological complexity associated with the transmission of this disease.

## 3. Diversity of Etiologic Agents of CBM

**Figure 1:**
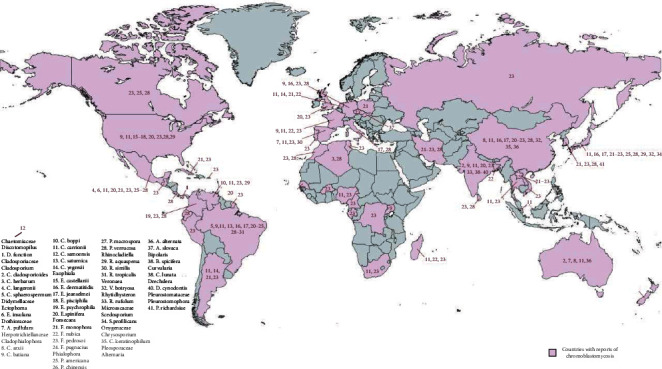
World distribution of fungal species responsible for CBM in humans. Each number on the map represents one species, shown in detail.

The fungi that cause CBM are dematiaceous and have melanin in the cell wall that interact with other proteins, lipids, and carbohydrates, making them more resistant to ultraviolet radiation, high temperature, and free radicals [19, 20]. These melanized fungi are saprophytes and are found in soil, plants, woods, and organic materials in decomposition [20]. Since the first description of CBM, different etiologic agents have been described, and according to the available data, we found that at least 41 fungal species cause CBM in humans, which are arranged in the following families: Chaetomiaceae, Cladosporiaceae, Didymellaceae, Dothioraceae, Herpotrichiellaceae, Hysteriaceae, Microascaceae, Onygenaceae, Pleosporaceae, and Pleurostomataceae. In addition to this diversity of species, genetic variation among strains distributed throughout the world may affect the outcome of infection [21]. In addition, one bacterial species mimics human CBM (Table 1). Fungal species have a wide geographic distribution (Figure 1); however, a high prevalence of CBM is found in the tropical and subtropical regions of the world [7, 22].

The Herpotrichiellaceae family has the most clinical importance [75] and includes the main genera that cause CBM: *Fonsecaea*, *Cladophialophora*, *Exophiala*, *Phialophora*, *Rhinocladiella*, and *Veronaea*.

**Figure 2:**
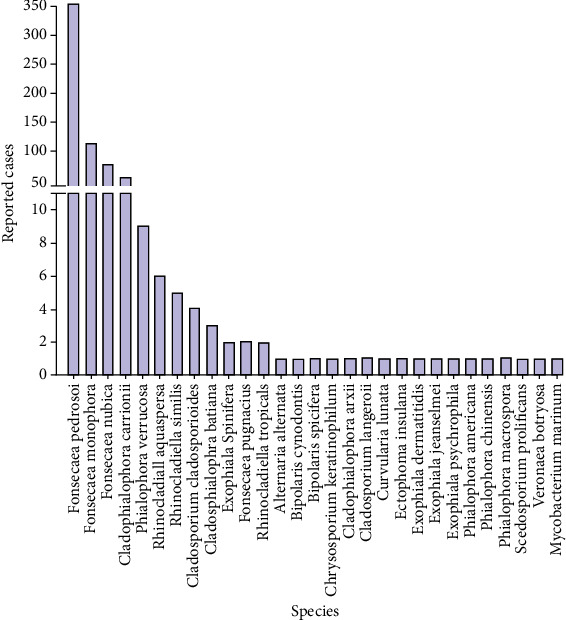
Distribution of human cases of CBM by etiologic agents. In the last 10 years, most published works identified the genus *Fonsecaea* and the following species *F*. *pedrosoi*, *F*. *monophora*, and *F*. *nubica* as the main etiologic agents of CBM. The *Cladophialophora* genus (*C. carrionii*) is the second most common affecting humans.

According to data available in the MEDLINE database, in the last 10 years, most of the cases were caused by fungi of the genera *Fonsecaea* and *Cladophialophora*, and the most prevalent species infecting humans were *F. pedrosoi* (353 reported cases), *F. monophora* (113 reported cases), *F. nubica* (75 reported cases), and *Cladophialophora carrionii* (53 reported cases) as shown in Figure 2, which, in fact, corroborate previously published works [75, 76].

In culture, Herpotrichiellaceae colonies, the most relevant family that causes human cases, show a grayish, blackish, or greenish color, and the identification of the genera is carried out according to morphology and type of reproduction [15]. The colonies of the genus *Fonsecaea* exhibit a greenish-grayish color, the hyphae are regular and branched at the apex, and the conidia are arranged in short chains; species belonging to the genus *Cladophialophora* also exhibit a greenish-grayish color, but the conidia are branched and arranged in long chains. Species from the genus *Exophiala* display black colonies and grow slowly, and the conodiophore is annellidic and shows intense reproduction by budding. In *Phialophora* colonies, they show a black-olivaceous color, and the conidia are produced in collarettes; in *Rhinocladiella* colonies, they show a blackish-greenish color, and the conidia occur in long sympodial cellular extensions. *Veronaea* colonies exhibit a blackish-brown color with green edges, and conidia also occur in long cellular sympodial extensions [15, 66]. It is not possible to differentiate species using only the morphological features of colonies or conidia; molecular techniques are essential to identify the infecting species. Furthermore, species of this family can develop in the tissues of humans and other groups of animals such as insects [70, 77, 78], amphibians [79, 80], rodents [81–83], felines [79], and caprines [85].

In addition to the Herpotrichiellaceaea family, another important family is *Cladosporiaceae,* and the most important species associated with CBM in animals and humans [86] are *C*. *cladosporioides* [72], *C*. *herbarum* [25], *C*. *langeronii* [26], and *C*. *sphaerospermum* [27]. In culture, colonies of this genus assume a grayish-greenish color [87], and conidia appear as branched chains and are adapted to spread over long distances [88]. These species exhibit a cosmopolitan distribution and can be found in the soil, air, tissues, food, and organic material [87, 88]. *C. cladosporioides* and *C. herbarum* are saprophytes and widely distributed, but other species, such as *C. sphaerospermum*, are saprophytes and live in harsh environments; others are phytophilous and fungicolous [88].

In the *Dothioraceae* family, only the *Aureobasidium pullulans* species is associated with CBM; human infection with this species is considered opportunistic and rare [29]. This fungus can not only be found in plant tissues but also in fresh and seawater from tropical and temperate regions of the world [29, 89, 90]. Initially, colonies assume a yellowish-brownish color and, over time, become grayish-greenish [89].

Within the Hysteraceae family, *Rhytidhysteron rufulum* is the species that is not only associated with diseases in plants but also causes CBM in immunosuppressed and immunocompetent humans [67, 91, 92]. At the beginning of the culture, the colonies display a light gray-greenish color and change from gray to dark green, presenting disorganized hyphae. In the Didymellaceae family, at least 10 species of fungi can infect humans, and the *Ectophoma insulana* species was characterized as an etiologic agent of human CBM [28]. Species of this genus can be found in soil, organic material, and water; additionally, these fungi parasitize invertebrates and vertebrates, but fungicolous species have been described [28, 93]. In culture, the colonies of the fungus exhibit a blackish-olive color [94].

The Pleosporaceae family has four genera of fungi with the potential to cause CBM in humans, as follows: *Alternaria slovaca* [95], *Alternaria alternata* [70], *Drechslera cynodontis* [24], *Bipolaris spicifera*, and *Curvularia lunata* [72]. This family has saprophytic taxon, as well as opportunistic species of humans [96, 97]. In culture, the genus *Alternaria* shows a gray-brown-black color, conidiophores exhibit few branches, and conidia are cylindric [98]. In culture, *Drechslera cynodontis* shows a grayish-blackish color, and multicellular conidia show sympodial development [24]. The genus *Curvularia* exhibits colonies with rapid growth and a brownish color [99]. In culture, the genus *Bipolaris* shows a light gray color at the beginning of the culture and, over time, a dark gray color is present [100].

The families Chaetomiaceae, Microascaceae, Onygenaceae, and Pleurostomataceae have the following etiologic agents that are capable of causing CBM in humans: *Dichotomopilus funicola* [23], *Scedosporium prolificans* [68], *Chrysosporium keratinophilum* [69], and *Pleurostomophora richardsiae* [73], respectively. Species in the genus *Chrysosporium* are saprophytes and can be found in soil, manure, and a rich environment in chitin, such as hair and feathers, rarely causing infection in humans [69]. Similarly, the species *P. richardsiae*, *D. funicola*, and *S. prolificans* are rarely implicated in human disease, and to our knowledge, they are considered opportunistic parasites in immunosuppressed patients [23, 73].

Classically, CBM is caused by fungi; however, the bacteria *Mycobacterium marinum* was shown to simulate CBM in humans [74, 101]. This species shows a cosmopolitan distribution and is found in fresh and sea water, including swimming pools and aquariums, and generally infects fish, amphibians, and reptiles; rarely, human infection is documented [74]. Disease begins after traumatic contact with animals infected with this bacteria, and immunosuppressed humans are particularly at risk [102].

As observed in Figure 1, a great biodiversity of species and the number of cases is concentrated between tropical and subtropical countries; it happens because tropical areas have ideal environmental conditions for the survival of fungi species, and possibly, human cases are condensed in some geographic areas more than others due to genetic mutations that make people more susceptible to fungal infections [103].

## 4. Modulation of Host Cells by Fungi and Fungi-Derived Antigens

The CBM infection begins after traumatic implantation of the filamentous forms of the fungus in the skin. Seven days after infection, the fungus is able to differentiate into muriform cells within phagocytic cells [4]. Muriform or sclerotic cells have a globe-shaped morphology and are multiseptated and pigmented cells.

The process of phagocytosis and fungi destruction may be suppressed due to the inability of Toll-like receptors to recognize the pathogen [104]. Although these specific data are already known, few studies have described the properties of virulence factors in modulation of host cells, and in this case, fungal melanin has been cited as an important molecule to protect the fungus in the intracellular environment. Melanin is a dark pigment found in all kingdoms, and, although not essential for survival, in fungi, it is related to the structure of the cell wall and pathogenicity. Indeed, this pigment has unpaired electrons in its structure that are capable of scavenging reactive species of oxygen and nitrogen, protecting muriform fungal cells in the harsh environment from macrophages and neutrophils [105, 106], and melanin suppresses phagocytosis [107].

Other antigens were involved in host cell adhesion, invasion, and survival in the host. It was shown that ectophosphatase of *R. aquaspersa* was involved in cell adhesion in epithelial cells, but it was reversed when conidia were previously treated with orthovanadate, molybdate, and antiphosphatase antibody [108]. Aspartic peptidase of *F. pedrosoi* was another antigen implicated in albumin and laminin cleavage and was associated with the dissemination and invasion of muriform cells throughout tissues [109]. Some intracellular microorganisms infect host cells with the aid of phospholipase enzymes [110, 111], and in CBM caused by *C. carrionii* and *F. pedrosoi*, an association was found between high phospholipase activity and severity of lesions [112]; possibly, the presence of an active phospholipase allows muriform cells to invade more cells by digestion of phospholipids from the cell membrane [113]. Furthermore, phospholipases are involved in the conversion of arachidonate from cell membranes to prostaglandins, and especially, prostaglandin E_2_ is able to downregulate macrophage activity, allowing intracellular microbes to survive [110].

**Figure 3:**
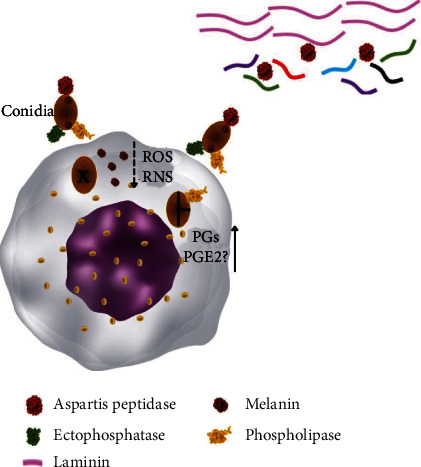
Interaction between the etiologic agents of CBM and phagocytic cells. The ectophosphatase enzyme is involved in the cell adhesion of conidia in the cell membrane of cells, and phospholipase may aid the process of cell invasion through digestion of lipids. In the intracellular environment, conidia differentiate into muriform cells, and released melanin with unpaired electrons has the ability to inhibit phagocytosis as well as scavenger ROS and NOS. Furthermore, some types of phospholipases induce the production of prostaglandins (PG), such as prostaglandin E2 (PGE2), which has a suppressive effect on macrophages. Extracellular fungi (such as muriform cells or conidia) secrete aspartic peptidase that is capable of cleaving laminin in connective tissues, ensuring that fungi spread through tissues.

Different species of fungi can cause CBM in humans, and possibly, each one has specific classes of molecules that may take some specific role during the process of infection; however, description about the antigens responsible for the maintenance of these fungi in humans may be useful as a molecular basis to understand the relationship between these pathogens and the hosts. Therefore, considering the available data on antigens from CBM etiologic agents and their interaction with host cells, we proposed a possible mechanism of infection and the spread of fungi in host tissues (Figure 3). Possibly, ectophosphatase, as well as other pathogen-associated molecular markers [114, 115], binds to the surface of the host cell, and in the intracellular environment, melanin suppresses reactive oxygen and nitrogen species, allowing fungal survival. Melanin may suppress the process of phagocytosis, and extracellular fungi, through the action of aspartate peptidase, are able to spread through tissues, ensuring parasite survival. Fungi phospholipases can aid in lipid digestion in the cell membrane, which should facilitate the process of infection. Furthermore, such enzymes, especially phospholipase E_2_, convert arachidonate into prostaglandins; some of them, such as prostaglandin E_2_, exhibit suppressive properties in macrophages, helping the persistence of muriform cells in host cells.

On the other hand, other fungal molecules can also play a role as virulence factors during CBM infection [116]; however, few data are available about them. Furthermore, the differentiation of hyphae into muriform cells contributes to the virulence of these fungi, since muriform cells exhibit a thicker cell wall than hyphae and it may contribute to survival in mammalian hosts, as demonstrated during the evolution of infection in mice infected with muriform cells of *F. pedrosoi*. In this situation, conidia and hyphae have been shown to induce self-healing disease, while muriform cells induce chronic disease [117] and the development of fibrous tissue [118].

## 5. Immune Response in CBM

Epithelial barriers protect organisms from infections, environmental damage, and dehydration. The breakdown of these natural barriers leads to remarkable changes in the morphology, physiology, and immune responses of injured tissue.

Upon damage, tissue and immune cells release metabolites, chemokines, and cytokines that will increase the permeability of the endothelium; moreover, immunological mediators along with the phenotypical changes that occur in the endothelium will allow cell leukocyte adhesion and migration, passage of plasma components into damaged tissue, and activation of immune cells. Therefore, an inflammatory environment would be established at the site of entrance of the etiologic agent of CBM, and in this specific case, the components of the innate immune response will first interact with the pathogen.

A special group of proteins reach the injured tissues after traumatic implantation of the fungus hyphae. These proteins belong to the innate system and are the so-called complement system proteins and play an important role during infectious and inflammatory processes. This system can be activated by three different pathways; the classical activation is initiated by binding of the antigen and antibody. The alternative activation pathway occurs when the C3b protein binds directly to the surface of the microbe. In the lectin pathway of complement system activation, some proteins from the complement system bind to pathogen-associated molecular patterns, mainly carbohydrate structure on the microbe cell membrane [119]. All of these pathways converge to the activation of the C3b molecule, which plays an important role in pathogen opsonization.

**Figure 4:**
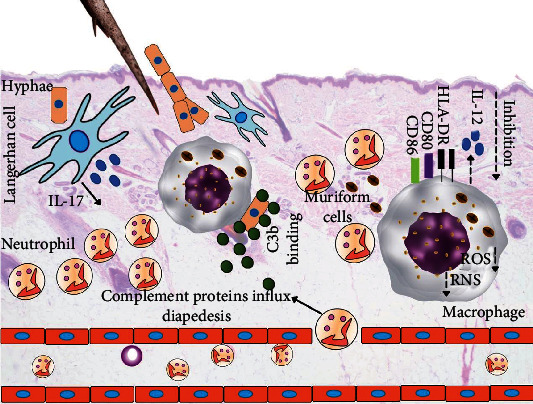
A prototype scheme of induction of innate immunity in chromoblastomycosis. Upon a traumatic injury with sharp objects contaminated with hyphae forms of pathogenic fungi, morphological and phenotypic changes occur in the endothelium, allowing the influx of inflammatory mediators, such as complement system proteins and neutrophils. C3b-opsonized hyphae are phagocytosed by macrophages; however, differentiation into muriform cells prevents the destruction of fungi. Furthermore, muriform cells and their antigens inhibit the expression of MHC class II and the costimulatory molecules (CD80 and CD86) of macrophages and dendritic cells. Production of IL-12 cytokine, NOS, and ROS is suppressed. Inhibition of these molecules alters the communication between innate and acquired immune responses. Additionally, the accumulation of antigens in dendritic cells favors the production of IL-17 cytokines, which attracts a second wave of neutrophils to the site of infection. Although neutrophils phagocytosed muriform cells, the fungi are resistant to destruction caused by the microbicidal molecules of such cells.

In this regard, *F. pedrosoi* has been found to be able to convert C3 and generate the chemoattractant C5a factor upon contact with human serum, suggesting that this specie of fungus activates the alternative complement pathway [120]. A further study corroborates it and showed that pigmented and hypopigmented mycelial forms of *F. pedrosoi* can activate the complement system, suggesting that at the moment of fungus infection through a traumatic injury, the complement system can be activated [121], opsonizing the fungi (Figure 4) and activating the local inflammatory response.

In fact, in a retrospective study by Uribe and collaborators, neutrophils, mononuclear phagocytic cells, and lymphocytes were demonstrated to be the most common cell types in the inflammatory infiltrate of patients with CBM, organized concentrically around muriform cells [122], as demonstrated in Figure 4. Although this study analyzed chronic cases of CBM, it is possible that activation of complement factors can attract cells of innate immunity to the site of infection, and immune cells, such as neutrophils, appeared to eliminate or attack *F. pedrosoi* muriform cells in the dermis [123]; however, neutrophils do not eliminate intraepidermal fungal elements [124]. Although experimental models of CBM caused by *F. pedrosoi* or *F. monophora* are self-limited, the importance of neutrophils in the elimination of these fungi was observed and possibly involves the interaction of fungus-associated molecular patterns, Toll-like receptors (TLR) 2 and 4, and the neutrophil extracellular traps [125–128].

In histological studies, macrophages have also been observed in the skin of patients infected with *F. pedrosoi*, and after phagocytosis, muriform cells are contained in cytoplasmic vacuoles. Although ultrastructural analysis by scanning electron microscopy suggested minor to moderate changes in the outermost layers of the cell walls of muriform cells, only a small number of yeasts showed signs of irreversible degeneration [129]. Furthermore, *in vitro* experiments showed that activated macrophages from Swiss mice inhibit or delay the transformation of conidia of *F. pedrosoi* into hyphae, although these macrophages were unable to kill intracellular fungi even in the presence of an oxidative response [105]. Although macrophages were able to produce hydrogen peroxidase in response to *F. pedrosoi*, possibly, fungal melanin scavenges this reactive oxygen species. Furthermore, it was shown that IFN-*γ*- and LPS-stimulated macrophages lost the ability to produce nitric oxide (NO) when incubated with *F. pedrosoi* melanin [130], and inhibition of NO production may be a pathogenic mechanism associated with fungal persistence in humans (Figure 4).

Although studies have focused on the influence of *F. pedrosoi* on the host cell, other species of the etiologic agent of CBM have been shown to induce different responses in macrophages. In a comparative study, it was shown that macrophage phagocytosis indexes were higher during infection with *F. pedrosoi* and *R. aquaspersa* compared to *C. carrionii* and *P. verrucosa*, and although macrophages had produced more NO and eliminated some intracellular *R. aquaspersa*, over time, few changes in the phagocytic index were detected, suggesting that CBM etiologic agents are resistant to microbicidal molecules produced by macrophages. This mechanism had a great impact on the expression of MHC class II and the costimulatory molecule CD80 [131], suggesting that the acquired immune response is suppressed. In fact, murine macrophages infected with *F. monophora* isolated from a patient with CBM produce low amounts of IL-12 cytokine [107] that may impact the development of a Th1 immune response. In humans with CBM, a similar mechanism may occur and should account for the chronicity of skin lesions; in this case, antigen-presenting cells, such as macrophages, Langerhan cells, and factor XIIIa^+^ dermal dendrocytes, were observed to accumulate *F. pedrosoi* antigens [132], which can alter communication between cells of innate and acquired immunity (Figure 4).

As stated above, each species may trigger a specific immune response in the vertebrate host, however, with the available data in the literature, this prototype mechanism of immunity is proposed as one possible pattern of immunity that can take place in the skin upon infection (Figure 4).

**Figure 5:**
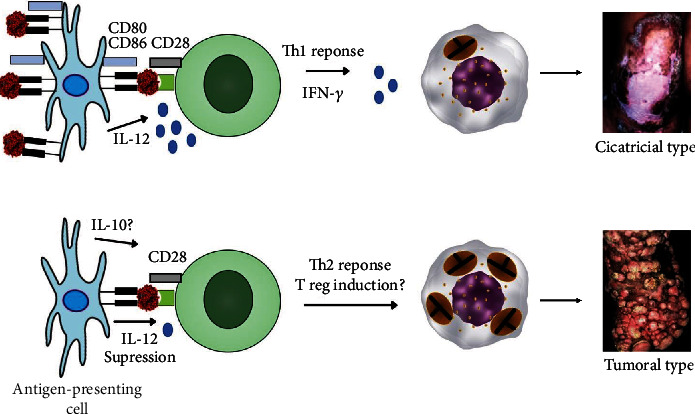
Acquired immune responses and outcome of CBM infection. Communication between APC and CD4 T cells involving MHC-TCR; costimulatory molecules (CD80/86–CD28) and IL-12 drive the development of a Th1 immune response and mild forms of CBM, such as the atrophic clinical type. On the contrary, the low expression of MHC class II associated with a lack of costimulatory response and low levels of IL-12 drives the immune response to a Th2 immune response, muriform cell multiplication, and disease development.

In fact, studies with patients with severe cases of CBM showed that the proliferative response of peripheral blood mononuclear cells (PBMC) stimulated with *F. pedrosoi* antigens were inhibited as well as the production of IFN-*γ*; in contrast, PBMC purified from the blood of patients with mild forms of CBM, defined as a solitary plaque or nodular lesion of less than 5 cm, proliferated and produced high levels of IFN-*γ* [133]; furthermore, some impairment in the communication of antigen-presenting cells and lymphocytes has been found in severe cases of CBM, since human macrophages expressed only basal levels of HLA-DR^+^, CD80, and CD86 molecules [134], suggesting that the strain, time of infection, and severity of the disease are critical factors in suppressing the both innate and acquired immune responses of patients with CBM, possibly caused by an excessive production of IL-10 (Figure 5). Furthermore, experimental evidence suggested that CD4^+^ T lymphocyte activity may have a greater impact on the development and chronicity of CBM [135]. In this specific case, CBM can be characterized as a spectral bipolar disease, and when a CD4^+^ Th1 immune response tends to develop, atrophic skin lesions can be observed in patients with CBM, but patients that mount a CD4^+^ Th2 immune response (Figure 5) show severe types of lesions, such as tumoral and verrucous types [136].

Although the accumulated knowledge on immunity in CBM has focused on the Th1 and Th2 paradigms, some studies observed the participation of the IL-17 cytokine in this fungal infection. In this case, IL-17-producing cells were detected at high densities by immunohistochemistry in the skin of patients with CBM. The main source of IL-17 is CD4^+^ T lymphocytes; however, other cell populations can produce it, such as IL-17-producing CD8^+^ T cells (Tc17), gamma-delta (*γδ*) T cells, and CD207^+^ cells (Langerhan cells) in the epidermis [137–139]. During infections with infectious agents, IL-17 recruits neutrophils (Figure 4), which can aid in the destruction of pathogens. In fact, a significant accumulation of neutrophils has been observed in the skin of experimental animals and humans with CBM and this finding is correlated with a high level of IL-17 *in situ*. In different fungal infections, neutrophils are capable of destroying fungi [140]; however, the etiologic agents of CBM may be resistant to the main microbicidal effects of these cells [141]. Thus, IL-17-producing cells may be involved in the persistence of fungi; however, more scientific evidence is needed to understand the real role of the IL-17 cytokine in CBM.

The humoral immune response during CBM infection has been poorly investigated. However, some works have already shown that antibodies may be an alternative to identify patients with CBM [142–144]; furthermore, specific IgG levels and subclasses can have a direct association with the severity of the disease [144]. It has been demonstrated that some antigens, such as fungal glucosylceramides and melanin, are immunogenic and antibodies produced in response to these antigens are capable of inhibiting the growth of different forms of *F. pedrosoi* [145, 146], suggesting a protective role *in vivo*. Despite the functional role of antibodies, to the best of our knowledge, the interaction between the etiologic agents of CBM and B cells is scarce in the literature.

## 6. Clinical and Epidemiology Aspects of CBM

The hyphae of the etiologic agents of CBM exist in the soil, plants, thorns, and even in the buccal apparatus and stings of invertebrates [147, 148]. In a traumatic event with such elements, hyphae can be inoculated through the skin, initiating the infection. Such ways of acquiring infection contribute to the occupational characteristic of the disease due to the high incidence in agricultural workers [149]. The period of incubation is unknown, and the evolution of the disease is slow and progressive. In most cases, the lower limbs are frequently affected areas, followed by the upper members, and the cephalic segments are rarely affected by the disease. Lesions are painless, unless there are secondary bacterial infections.

The initial lesion appears at the site of hyphae inoculation; however, some patients did not report traumatic lesions at the site of lesion development. Initially, an isolated macular lesion appears and evolves to an erythematous papule, which gradually increases in size and further develops into a papulosquomous form, sometimes with a polymorphic aspect that can be misdiagnosed with other skin diseases. Progressively, the lesions become the characteristic verrucous aspect. At the beginning of the disease, the lesions are asymptomatic and did not interfere with patient activities, but over time, patients reported itching, accompanied or not by pain [3].

Skin lesions develop in extension and may be a consequence of scratching or autoinoculation of the satellite lesion. Additionally, fungi can spread through tissues by the lymphatic system [150]. Clinical changes progress slowly, producing fibrotic changes in the skin and lymphatic stasis, similar to elephantiasis due to lymphatic edema and damage to lymphatic vessels [15]. Infection may occur anywhere in the body, but if it starts in the lower limbs, the lesions tend to spread to the knee, thigh, or instep [151].

**Figure 6:**
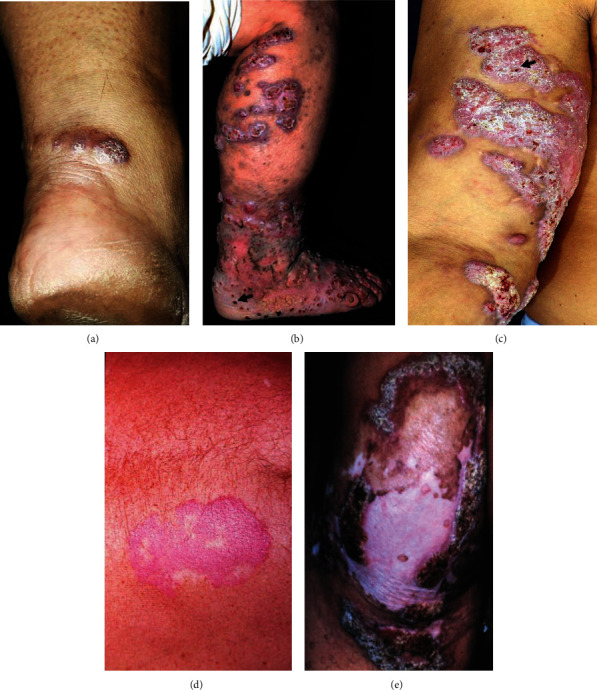
Five different clinical forms can be identified in patients with CBM, such as nodular (a), tumoral type (b), verrucous (c), plaque (d), and cicatricial (e) forms. The black arrow shows black dots in the lesions.

CBM exhibits five different clinical forms: nodular, tumoral type, verrucous, plaque, and cicatricial (Figure 6). In the nodular form, fairly soft, pale pink or purple, small nodules can be identified, and the surface of the nodules may be smooth, papillary, or scaly. If not properly treated, these nodules can gradually develop into the tumoral form of the CBM. In the tumoral form, the lesions are larger and more protruding, papillomatous, or lobulated than in the nodular form and can exhibit tumor-like masses. Additionally, these lesions can be partially or completely covered with epidermal remains, scabs, and black dots. In the verrucous clinical form, the lesions resemble warts and the main characteristic of this type of lesion is the presence of hyperkeratosis and black dots on the top of the skin lesions. The plaque type is the most uncommon clinical form of CBM, and the lesions are planoconvex with various shapes and sizes, and the surfaces are scaly with a reddish to violet color. In the cicatricial clinical form, lesion develops by the peripheral growth with atrophic scarring, and in the middle of a lesion, a healing process occurs [76]. In addition to the traditional classification of CBM, Badali and collaborators identified a possible new clinical form of CBM that was caused by hyphae of *R. aquaspersa* and resembled botryomycosis [63].

Additionally, to this classification, CBM can be classified into three levels according to the severity of the disease: (1) In the mild form, single scales or nodules have up to 5 cm of diameter; (2) in the moderate form, single or multiple lesions of tumoral, verrucous, or plaque types isolated or conjoined covering one or two adjacent areas of the body exhibit up to 15 cm of diameter; and (3) the severe form includes any type of single or multiple lesions covering extensive skin areas. However, to assess the severity of the disease, Castro and Andrade take into account the size and number of lesions, the presence of complications, and the response to previous treatments [2].

In general, systemic involvement in CBM is not observed and blood biochemical parameters are normal. Eventually, in long-standing lesions, carcinomatous transformation may occur, although such tumors exhibit slow progression and have a noninvasive profile [152]; however, some tumors may show an invasive profile and can be the result of long-standing chronic inflammation due to the presence of muriform cells [153].

CBM is common in tropical and subtropical countries; however, records of this infection have been described in temperate countries [3]. In these areas, the disease is prevalent in men, between 30 and50 years of age, working in agriculture. In adolescents, this disease is rare. The record of a traumatic event is not often informed, but it has been assumed that traumatic implantation of the etiologic agent is the main cause of infection. The lower extremities are the most affected area, followed by the arms and the cephalic segment [22, 104, 154].

In the African continent, CBM is predominant in Madagascar and South Africa; in Asia, it occurs in India, China, and Japan; however, reports have also been observed in Australia. In South America, Brazil, Mexico, and Venezuela are the most important countries with the occurrence of CBM [155]. In the USA and Europe, CBM is rare [156].

## 7. Diagnosis and Differential Diagnosis

The morphology of CBM lesions resembles the following infectious diseases: lobomycosis, sporotrichosis, phaeohyphomycosis, protothecosis, tuberculosis verrucose, leishmaniasis, hanseniasis, eumycetoma, and botryomycosis, as well as other noninfectious diseases such as carcinoma, ceratoacantoma, lupus erythematous, and cutaneous sarcoidosis. Therefore, the clinical aspect of lesions, localization, patient record, and disease evolution are important factors to investigate and may help with differential diagnosis.

The diagnosis of CBM is based on clinical and epidemiological data; however, it should be confirmed by the demonstration of the etiologic agent in the lesion. Visualization of the etiologic agent is performed by direct mycological tests on skin scrapings containing crusts, clarified by potassium hydroxide. This preparation allows the identification of muriform cells, which have 10 to 14 *μ*m of diameter and exhibit a brownish color with round-ellipsoid shape; they are crossed by transverse and longitudinal septa and show a thick cell wall. This finding is typical of CBM, and such forms are also called sclerotic bodies, fumagoid bodies, or Medlar bodies. To perform direct observation, the biological material should be collected in black dots. To cultivate the etiologic agent, areas without signs of secondary infection should be collected because bacteria in the culture medium can inhibit fungi growth. The morphology and color of the colonies along with microscopic aspects will be useful to identify the genus of the fungus, but molecular biology techniques are required to identify species [157].

The biopsy should be taken in tissues without areas of necrosis or loose aspect. In histological sections of the skin stained with hematoxylin and eosin, it is possible to observe irregular achantosis or pseudoepitheliomatous hyperplasia in the epidermis. A lymphohistiocytic inflammatory infiltrate characterized by the presence of granulomas, neutrophils, giant cells, plasma cells, and isolated or grouped muriform cells can be observed in microabscesses with a brownish color [3]. In the tissues muriform cells predominate, however, it is possible to observe hyphae in the dermis [15, 158]. Muriform cells can also be observed in the epidermis due to transepidermic elimination of the fungi. PAS and Grocott-Gomori stains can be used in the histological section as specific stains for fungi; however, they did not increase diagnostic sensitivity, as happens in phaeohyphomycosis [151]. On the contrary, the background of both stains can be considered a major drawback, leading to misdiagnosis of CBM. Eventually, Ziehl-Neelsen and Wade-Fite stains may be used to observe muriform cells [158].

Although morphological observation of the etiologic agent represents an important tool to diagnose CBM, it is important to note that the precision of the technician in the identification of the genus is essential to direct therapeutic approaches as well as the outcome of the disease. In contrast, misdiagnosis of cutaneous mycosis frequently worsens the clinical outcome of the disease because it may employ drugs or approaches [159] that are not fully active in the infecting fungus [160].

## 8. Immunological Diagnosis

Immunological diagnosis (serology and delayed-type hypersensitivity (DTH)) is no longer used in the diagnosis of CBM, considering the high sensitivity of mycological and histopathological diagnosis. However, some works demonstrated that DTH using chromomycin exhibited 90% of positivity and 98.8% of specificity among patients with CBM caused by *F. pedrosoi* [161]. The somatic antigen produced with *C. carrionii* was analyzed in an ELISA assay during the therapeutic evolution in patients with CBM caused by *C. carrionii*. In this study, 100% of the serum of patients with CBM was shown to react to this antigen before treatment, but the serum of healthy people did not react, suggesting that it is an important tool for diagnosing active disease [162]. Furthermore, it was verified by immunoblotting that an antigenic fraction of 54 kDa purified from *F. pedrosoi* reacted positively in 96.7% when incubated with serum from patients with CBM caused by *F. pedrosoi*, and, importantly, this antigen did not cross-react with serum from patients with sporotrichosis, leishmaniasis, or healthy people [163].

## 9. Treatment of CBM

**Figure 7:**
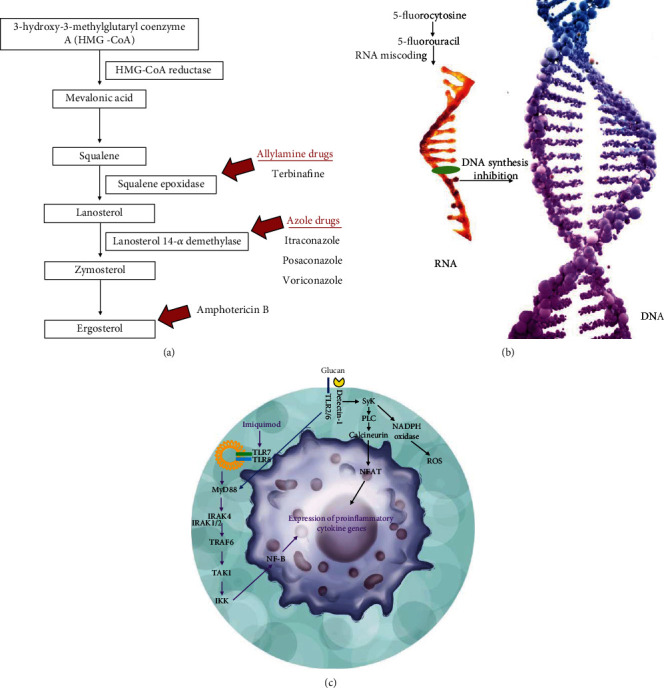
Drugs used in the treatment of CBM. (a) This scheme shows the biosynthesis of ergosterol, which is one of the main components of cell membranes of fungi, as well as enzymes that common drugs target, interrupting the production of ergosterol. (b) Some antifungal drugs, such as 5-fluorocytosine, inhibit DNA synthesis in etiologic agents of CBM. (c) Some molecules, such as imiquimod and glucan, have been used to stimulate the immune response of hosts; imiquimod is an agonist of TLR7/8 and glucan in a pathogen-associated molecular pattern capable of binding to the TLR2/6 and detectin-1 receptor. In both cases, such molecules trigger the production of proinflammatory cytokines from the Th1 immune pattern.

Antifungal drugs usually target specific molecules of the pathogen or interfere with biosynthetic pathways, inhibiting the production of a final product (Figure 7(a)). The polyene class of antifungal drugs, such as amphotericin B and nystatin, is amphipathic with a hydrophobic polyene hydrocarbon chain and a hydropholic polyhydroxyl chain. In fungi, amphotericin B intercalates with ergosterol in the cell membrane, forming channels that cause leakage of fungal cell components and death. Amphotericin B, as well as nystatin, has a broad spectrum of fungicidal activity; however, the main limitation is associated with the side effects induced in patients. In addition to the enhanced efficacy and low toxicity of the liposomal version of amphotericin B, the high costs limit its use in low-income countries.

In CBM, one of the first reports showing the activity of amphotericin B was published in 1959. The patient was a man, a farm worker in Porto Rico, and had CBM lesions for 22 years. No success was achieved with different medications or excision of the lesions. In New York City, *F. pedrosoi* was identified in the skin lesion and treated with a total of 60 mg of amphotericin B administered by local infiltration for four weeks. According to the authors, three months after treatment, the skin lesions healed, and six months after treatment, the skin biopsy and the skin culture were negative [164]. In another patient with CBM for 11 months, amphotericin B administered intralesionally using vibrapuncture was also effective in healing CBM lesions [165]. Although amphotericin B showed curative properties when administered locally, high doses can cause severe local pain, hemolysis, thrombosis, fibrosis, and gangrene [166]. In addition, some skin lesions were too large to choose a local treatment with amphotericin B, and the dose required to heal the lesions was too high to be used in systemic application, due to the high toxicity of amphotericin B in humans. Furthermore, *in vitro* studies suggested that amphotericin B would not be a good choice for the treatment of CBM, considering that it exhibited less activity than the following antifungal drugs posaconazole, itraconazole, isavuconazole, and voriconazole in clinical isolates of *F. pedrosoi*, *F. monophora*, and *F. nubica* [167].

Another widely used antifungal drug in patients with CBM is itraconazole, which belongs to the azole class of antifungal medications. All azole drugs have a heterocyclic moiety capable of binding to an iron atom in the heme group of the active site of the enzyme lanosterol 14-*α* demethylase (Figure 7(a)), inhibiting the demethylation of lanosterol and consequently the production of fungal ergosterol [168]. One of the first studies using itraconazole in CBM therapy showed that the efficacy may be related to the infecting species, as 89% of patients infected with *C. carrioni* and treated with 400 mg of itraconazole exhibited complete cure, while only 40% of patients infected with *F. pedrosoi* presented cure. Furthermore, the efficacy of itraconazole used as monotherapy in CBM had a direct association with the severity of skin lesions [169]. Furthermore, a Brazilian study reinforced that the severity of lesions caused by *F. pedrosoi* can be correlated with a low prognostic of cure and long-term treatment with itraconazole [57].

Despite the therapeutic activity of itraconazole in CBM therapy, monotherapy has been avoided and has often been associated with 5-fluorocytosine or terbinafine. The drug 5-fluorocytosine belongs to the class of pyrimidines and is a synthetic fluorinated analogue of cytosine. In the fungal cytoplasm, 5-flucytosine is deaminated and becomes 5-fluorouracil, which acts as an antimetabolite [170], causing RNA miscoding and inhibiting DNA synthesis (Figure 7(b)). The first cases of CBM treated with 5-flucytosine occurred in the early 1970s [31, 171]; however, the development of resistance was a concern and led to the abandonment of monotherapy with this drug. In contrast, 5-flucytosine at 50-150 mg/kg/day has recently been used in association with itraconazole at 100-400 mg/kg/day for the treatment of CBM [172, 173].

Terbinafine belongs to the class of allylamines that inhibit the fungal enzyme squalene-2,3-epoxidase, responsible for the conversion of squalene to lanosterol (Figure 7(a)). This inhibition affects the production of ergosterol as well as the cell membrane of fungi; additionally, inhibition of squalene epoxidase leads to accumulation of squalene, which is toxic to fungi, and causes a fungicidal effect [174]. In CBM, generally, terbinafine used along with itraconazole seems to speed-up the cure of CBM. The first trial showing the efficacy of terbinafine, used as monotherapy, was carried out in 1996, and it was demonstrated that 82.5% of the patients infected with *F. pedrosoi* under treatment with terbinafine by oral route at 500 mg/kg/day during 6-12 months exhibited complete regression of cutaneous lesions [175]. Possibly, this high efficacy was associated with the elevated fungicidal potential of this drug (*in vitro*) over the etiologic agents of CBM [41, 176]. In some patients, terbinafine interrupts the development of lesions; however, in others, cure is not observed [177, 178]. On the other hand, some studies showed that the association between itraconazole and terbinafine showed synergistic or additive effects when combined *in vitro* [179], which in fact can improve the cure rate in CBM.

In patients with CBM, oral therapy with itraconazole and terbinafine was shown to improve skin lesions in patients with long-term CBM (8-23 years) caused by *F. pedrosoi* without significant adverse side effects [180]. Other agents of CBM, such as *P. richardsiae*, were also eliminated when patients were treated with such an association after a few months of treatment [73], which in fact reinforces that both drugs can be used together in therapy; in addition, both drugs can play a role in the induction of the cellular immune response during the healing process of patients with CBM [181], supporting the fungal elimination process. In general, terbinafine and itraconazole are administered orally at the following doses 200-400 mg/kg/day and 250-500 mg/kg/day, respectively [57, 75, 180, 182]. However, it has been observed that the cure rate of this association fluctuates between 15 and 80% depending on the severity of the disease and the infecting species [3].

Posaconazole and voriconazole, members of triazole antifungal drugs (Figure 7(a)) have been used in CBM at 800 mg (daily administered orally divided in two or four doses) or 400 mg (daily, given orally divided in two doses), respectively. Both drugs were used in patients with long-term CBM lesions that were refractory to standard drugs. Posaconazole induced clinical cure in 5 of 6 patients; additionally, this drug was well tolerated during treatment, which lasted 174-376 days [183]. In three patients with extensive long-term CBM, and with a history of treatment failure, voriconazole improved skin lesions; however, when treatment was withdrawn, muriform cells were isolated from two patients [178]. Another study corroborated the therapeutic potential of voriconazole in a patient refractory to conventional treatment performed with itraconazole, terbinafine, itraconazole, thermotherapy, or cryosurgery, alone or in combination [184]. In fact, a systematic review highlights the great potential of voriconazole to eliminate some etiologic agents of CBM (*in vitro*) alone or in combination [185], reinforcing that this drug may be useful in the treatment of CBM or at least be the choice in limited cases, especially those refractory to conventional treatment. However, the high costs associated with long-term therapy limit the routine use of this drug, especially in low-income countries.

Chemotherapic treatment of CBM has mainly focused on the combination of conventional antifungal drugs; however, the cure rate is variable and depends on the extent of the lesion, the physiological status of the patient, and the infecting species. To avoid refractoriness of the disease to conventional drugs, small trials have been conducted in patients with CBM and show that the combination of classical antifungal drugs with immunomodulatory compounds is effective in the treatment of CBM. A patient with severe CBM caused by *F. pedrosoi* did not respond to the classical treatment carried out with itraconazole or with the association itraconazole plus terbinafine; however, a significant improvement was observed when the patient was treated during two years with itraconazole (400 mg/day) plus glucan (5 mg/week), a pathogen-associated molecular pattern [186], from the cell wall of *Saccharomyces cerevisiae*. Furthermore, a swift to a Th1 immune response was observed after 6 months of therapy [187], suggesting that in severe cases it would be relevant to combine antifungal and immunomodulatory drugs (Figure 7(c)).

Another immunomodulator recently introduced in CBM combined therapy is imiquimod. This drug is approved by the European Medicine Agency and the Food and Drug Administration for the treatment of actinic keratosis, human papillomavirus-induced genital warts, and superficial basal carcinoma. Imiquimod is a Toll-like receptor 7/8 agonist (Figure 7(c)), which can stimulate the immune response, through dendritic cell activation and macrophage polarization to the M1 subset, and in T cells, it can drive the immune response to a Th1 immune pole [188]. Therefore, imiquimod appears to be an interesting adjuvant for the treatment of intracellular pathogens, as has been shown in other infectious diseases [189] that the Th1 immune response is downregulated, as is the case of leishmaniasis [190]. The first study showed that imiquimod alone or associated with itraconazole or itraconazole plus terbinafine significantly improved the lesions of four patients with CBM caused by *F. pedrosoi* [191]. A further study showed that three patients with nonextensive CBM lesions caused by *F. pedrosoi* presented complete cure after imiquimod treatment and was associated with an active inflammatory process observed in the skin [192]. Although imiquimod has the ability to activate the immune system, it should be used in conjunction with classical antifungal drugs in extensive lesions.

Recently, it was verified that acitretin exhibited an interesting activity as an adjuvant drug in the treatment of patients with CBM. This drug is a synthetic retinoid and has been used in the treatment of psoriasis [193]. Acitretin binds to retinoic acid receptors, changing gene expression and triggering an antiproliferative response; thus, in disorders of keratinization, a normalization of epidermal cell proliferation, differentiation, and cornification will be observed. Acitretin plays a role in inhibiting endothelial growth and angiogenesis; in addition, it has been associated with suppression of inflammation [194, 195]. In CBM, excessive cell growth and keratinization is associated with thickening of the skin, plaque formation, and keratinization; thus, a drug capable of reducing keratinization and plaque thickness may allow better absorption of topical drugs. In a patient diagnosed with psoriasis and CBM caused by *F. monophora*, oral treatment with itraconazole did not improve skin lesions; however, when acitretin (20 mg/day) was added to treatment, the lesion resolved completely after the first month of treatment. Another study showed that the combination of acitretin, imiquimod, and itraconazole partially controlled the lesions of two patients with long-lasting CBM lesions [196]; possibly in such patients, acitretin inhibited skin keratinization, allowing imiquimod to permeate through the skin, which in turn activated immune cells that destroyed intracellular pathogens; and itraconazole, as an antifungal drug, eliminated muriform cells. Therefore, the action of these three drugs can be complementary in the treatment of long-standing and severe cases of CBM. Other molecules, such as tricyclazole, an inhibitor of melanin [197], HIV peptidase inhibitors [198], and 1,10-phenanthroline-5,6-dione [199], have also been studied in the context of CBM and show interesting fungicidal activity.

Most people with CBM get infected by working in agriculture, and sometimes, they treat the lesions using the local resources, such as medicinal plants. In this regard, it was observed that healers and health agents living in the district of Curituba, city of Canindé do São Francisco, Sergipe State, Brazil, use plants from the Caatinga biome to treat mycosis, and water infusion extract produced with the stem of *Ziziphus joazeiro* Mart. (Rhamnaceae) or leaves of *Caesalpinea pyramidalis* Tul. (Caesalpinaceae) eliminated *F. pedrosoi* [200], suggesting that bioactive molecules from plants used in popular medicine may represent an alternative to treat CBM. The species of plant *Pterocaulon alopecuroides* (Lam.) D.C. (Asteraceae), also used in popular medicine for the treatment of superficial mycosis, eliminated 23 strains belonging to the following species: *F. pedrosoi*, *F. compacta*, *C. carrionii*, *R. aquaspersa*, *P. verrucose*, and *E. jeanselmei* (*in vitro*) [201]. Although some plants have fungicidal potential over the etiological agents of CBM, up to now, no purified compounds have been isolated and characterized from these plants. However, it was demonstrated that the natural product ajoene, an organosulfur compound purified from garlic, showed therapeutic activity when applied topically in patients with CBM caused by *C. carrionii*; additionally, such activity was similar to 5-fluorouracil treatment [202, 203]. Possibly, ajoene inhibits the biosynthesis of phosphatidylcholine, a cell membrane component [204]. Although different plants have been studied in the context of skin diseases by traditional communities [205, 206], very few advances have been made in the field of CBM.

In addition to chemotherapy, some physical approaches can be used in the treatment of CBM, such as thermotherapy, photodynamic therapy, and surgery. Although helpful, physical approaches present limitations and depend mainly on the size and location of the lesions.

In the case of treatment using liquid nitrogen or heat, an important factor to consider is the size of skin lesions, and if the lesions were considered large, the therapy should be applied in sections. The rationale for cryotherapy is based on the fact that nitrogen applied to the skin will induce an inflammatory reaction and necrosis and consequently fungus elimination. However, this method exhibits high relapse indexes and often leaves achromic and unaesthetic scars [207]. In heat therapy, affected tissue will be heated above 46°C, that is, the highest temperature at which CBM pathogens grow. Although effective for some patients, this therapy can be used in association with first-line drugs in some patients [208].

Photodynamic therapy was developed for the treatment of cancer but has recently been used in CBM treatment. In this therapy, a photosensitizer is used, and upon the incidence of light in a specific wavelength, several chemical reactions lead to the production of microbicidal molecules, such as reactive oxygen and nitrogen species, that will damage cells as well as microorganisms. In CBM, different reports already showed the efficacy of this method, which was able to decrease the size of cutaneous lesions by 80-90% after the sixth application in ten patients [209]. Additionally, this technique has been used successfully in complicated and refractory cases of CBM [210]. This physical approach is indeed promising; however, the antimicrobial effects of photodynamic therapy seem to occur only when light is turned on [75]. Furthermore, it is considered an expensive treatment method and unfortunately may not be available in endemic areas.

Surgery is considered the best physical method to be employed in CBM. In this case, the skin lesion can be removed, followed or not by the insertion of a skin graft. Although the infection did not reach a deep tissue, such as the muscular fascia and muscles, it is highly recommended to perform a large excision of the affected area with 5 mm margins, avoiding the spread of the fungus through the lymphatic system [151]. Mohs micrographic surgery can also be used in limited cases [211]. In severe and extensive cases of CBM, the only known resource is amputation of part or all of the affected area, although relapses can still be observed.

## 10. Experimental CBM

Experimental models of infectious diseases frequently shed light on the immunological, pathological, biochemical, and cellular mechanisms that microbes subvert to infect vertebrate hosts. Furthermore, a reliable experimental model is useful to develop new therapeutic or immunoprophylactic approaches.

In CBM, different studies attempted to standardize an animal model to mimic the chronic form of this disease; however, the majority of them reproduced a self-healing model of CBM with the participation of hyphae and conidia forms [126, 212, 213], which differ from natural infection, where muriform cells are considered the parasitic forms. Such studies employed different species of hosts and fungus, number of inoculated entities, route of infection, and number of injections, but none of them reproduced a long-lasting infection.

However, some works standardized more stable experimental models of CBM that lasted at least six months. One of this study showed that *F. pedrosoi* cultured for a long period *in vitro* induced chronic lesions in BALB/c mice [214], although this study is important to the field, the production or standardization of an old colony may represent an obstacle to reproduce this experimental model. On the other hand, a progressive model of CBM was induced in athymic mice [215] and CD8 knockout mice [216]; although these models can be useful to depict the process of infection and differentiation of hyphae or conidia into muriform cells *in vivo*, as well as the development of new drugs, it may not be an interesting model to study the cellular immune response, as well as the potential of immunomodulatory drugs or vaccines in CBM.

These studies highlight the difficulty of standardizing a reproductible experimental model of CBM and at least in part justify the lack of studies characterizing vaccines, immunomodulatory molecules, and even studies on fungus and host relationship.

## 11. Challenges and Future Perspectives

CBM is caused by different species of dimorphic fungi that mainly affect people working in the field of agriculture and, in general, such people that live in low-income countries. Additionally, there is a limited number of drugs that can be used in the treatment of CBM, and although urgent, few studies have identified new molecules with action on the etiologic agents. In the field of chemotherapy, there is a real lack of clinical studies that intended to standardize a therapeutic scheme for the treatment of patients; on the contrary, studies have reported the efficacy of therapeutic schemes only in small groups of patients. Thus, the authors of this work suggest the development of projects on drug discovery for the treatment of CBM, which, in fact, is a huge open field to work on.

Few prophylactic measures can be performed to avoid infection and basically rely on the use of adequate wears and gloves during field work; however, it is important to consider that vulnerable people live in tropical countries and places that reach high temperatures during the summer, which makes the use of long wears during the day impracticable. Unfortunately, people cannot take advantage of an effective vaccine because it has not yet been developed. However, immunological studies conducted on the relationship of fungi and host suggest that an effective Th1 immune response is important for eliminating muriform cells. Studies of vaccination for other infectious diseases have already shown that different adjuvants can trigger a Th1 immune response and can be used in the field of CBM; even imiquimod, which was used as a topical drug in CBM, can be used as an immunological adjuvant. However, the lack of experimental models that mimic chronic CBM infection hampers the development of vaccines.

Another challenge related with CBM is the diagnosis that has been carried out based on morphological features and the identification of the etiologic agent by molecular biology techniques. Although such methodologies have been considered the gold standard for identifying infecting species in different superficial mycosis, only well-trained and experienced professionals are able to do it correctly; therefore, it is important to develop additional, accurate, affordable, and accessible methods for the diagnosis of CBM.

As observed, the majority of affected people live in isolated areas of low-income countries with limited access to medical center facilities. Thus, it is important to create government policies to bring basic information about CBM to these people, such as etiologic agents, form of transmission, importance to wear adequate clothing during work in the field, and more importantly, to perform a correct diagnosis and offer adequate treatment. Furthermore, funding agencies could create supporting programs devoted to prophylaxis, diagnosis, and treatment of CBM.

All these actions should be carried out together to inform, prevent, and treat affected people; otherwise, CBM will never be controlled or even extinct from the tropical and subtropical areas of the planet.

## Figures and Tables

**Table 1:** Human CBM is caused by 41 species of fungi and one species of bacterium that are distributed into several families. All of these species cause five different clinical forms of CBM. In addition, the bacteria *Mycobacterium marinum* (Mycobacteriaceae family) cause skin disease in humans that resembles CBM.

Family	Species	Clinical forms	References
Chaetomiaceae	*Dichotomopilus funicola*	Tumoral type	[23]
Cladosporiaceae	*Cladosporium cladosporioides*	Nodular, verrucous, plaque	[24]
	*Cladosporium herbarum*	Nodular, verrucous	[25]
	*Cladosporium langeronii*	Tumoral type	[26]
	*Cladosporium sphaerospermum*	Not available	[27]
Didymellaceae	*Ectophoma insulana*	Nodular, verrucous, plaque	[28]
Dothioraceae	*Aureobasidium pullulans*	Nodular, verrucous	[29]
Herpotrichiellaceae	*Cladophialophora arxii*	Nodular	[30]
	*Cladophialophora batiana*	Nodular, plaque	[31, 32]
	*Cladophialophora boppii*	Nodular, plaque	[33]
	*Cladophialophora carrionii*	Nodular, tumoral type, verrucous, plaque, cicatricial	[34–38]
	*Cladophialophora samoensis*	Not available	[39]
	*Cladophialophora saturnica*	Nodular	[40]
	*Cladophialophora yegresii*	Not available	[41, 42]
	*Exophiala castellanii*	Not available	[43]
	*Exophiala dermatitidis*	Nodular	[44]
	*Exophiala jeanselmei*	Nodular, verrucous, plaque, tumoral type	[45–47]
	*Exophiala pisciphila*	Verrucous	[48]
	*Exophiala psychrophila*	Verrucous, plaque	[49]
	*Exophiala spinifera*	Nodular, verrucous, plaque	[50, 51]
	*Fonsecaea monophora*	Tumoral type, verrucous, plaque	[52–54]
	*Fonsecaea nubica*	Nodular, tumoral type, verrucous, plaque, cicatricial	[55, 56]
	*Fonsecaea pedrosoi*	Nodular, tumoral type, verrucous, plaque, cicatricial	[55, 57]
	*Fonsecaea pugnacius*	Verrucous, plaque	[58]
	*Phialophora americana*	Nodular, verrucous, plaque	[59]
	*Phialophora chinensis*	Plaque	[59]
	*Phialophora macrospora*	Nodular, verrucous, plaque	[59]
	*Phialophora verrucosa*	Nodular, verrucous, plaque	[60, 61]
	*Rhinocladiella aquaspersa*	Plaque, verrucous	[62, 63]
	*Rhinocladiella similis*	Nodular, tumoral type, verrucous, plaque	[55, 64]
	*Rhinocladiella tropicalis*	Plaque	[65]
	*Veronaea botryosa*	Nodular, verrucous, plaque	[66]
Hysteriaceae	*Rhytidhysteron rufulum*	Nodular	[67]
Microascaceae	*Scedosporium prolificans*	Nodular	[68]
Onygenaceae	*Chrysosporium keratinophilum*	Nodular, verrucous, plaque	[69]
Pleosporaceae	*Alternaria alternata*	Verrucous, plaque	[70]
	*Alternaria slovaca*	Not available	[71]
	*Bipolaris spicifera*	Not available	[72]
	*Curvularia lunata*	Not available	[72]
	*Drechslera cynodontis*	Verrucous, plaque	[24]
Pleurostomataceae	*Pleurostomophora richardsiae*	Nodular, verrucous, plaque	[73]
Mycobacteriaceae	*Mycobacterium marinum*	Verrucous, plaque	[74]

## Data Availability

No data were used to support this study.
